# Establishing network pharmacology between natural polyphenols and Alzheimer’s disease using bioinformatic tools – An advancement in Alzheimer’s research

**DOI:** 10.1016/j.toxrep.2024.101715

**Published:** 2024-08-23

**Authors:** Arunkumar Subramanian, T. Tamilanban, Vetriselvan Subramaniyan, Mahendran Sekar, Vipin Kumar, Ashok Kumar Janakiraman, Saminathan Kayarohanam

**Affiliations:** a Department of Pharmacology, SRM College of Pharmacy, SRM Institute of Science and Technology, Kattankulathur, Chengalpattu, Tamilnadu 603203, India; b Department of Medical Sciences, School of Medical and Life Sciences, Sunway University Jalan University, Bandar Sunway, Selangor Darul Ehsan 47500, Malaysia; c School of Pharmacy, Monash University Malaysia, Bandar Sunway, Subang Jaya, Selangor 47500, Malaysia; d Department of Pharmaceutical Sciences, Gurukul Kangri (Deemed to be University), Haridwar 249404, India; e Faculty of Pharmaceutical Sciences, UCSI University, Cheras, Kuala Lumpur 56000, Malaysia; f Faculty of Bioeconomics and Health Sciences, University Geomatika Malaysia, Kuala Lumpur 54200, Malaysia

**Keywords:** Alzheimer’s disease, Polyphenols, Insilico, Network pharmacology

## Abstract

Alzheimer’s disease (AD) is a major cause of disability and one of the top causes of mortality globally. AD remains a major public health challenge due to its prevalence, impact on patients and caregivers, and the current lack of a cure. In recent years, polyphenols have garnered attention for their potential therapeutic effects on AD. The objective of the study was to establish network pharmacology between selected polyphenols of plant origin and AD. Insilico tools such as SwissADME, ProTox3.0, pkCSM, Swiss Target Prediction, DisGeNET, InterActiVenn, DAVID database, STRING database, Cytoscape/CytoHubba were employed to establish the multi-target potential of the polyphenolic compounds. The present study revealed that out of 17 polyphenols, 10 ligands were found to possess a drug-likeness nature along with desirable pharmacokinetic parameters and a lesser toxicity profile. Also, the results highlighted the possible interactions between the polyphenols and the disease targets involved in AD. Further, this study has shed light on the mTOR pathway and its impact on AD through the autophagic mechanism. Overall, this study indicated that polyphenols could be a better therapeutic option for treating AD. Hence, the consumption of polyphenolic cocktails as a part of the diet could produce more effective outcomes against the disease. Additional studies are warranted in the future to explore additional pathways and genes to provide a comprehensive understanding regarding the usage of the shortlisted polyphenols and their derivatives for the prevention and treatment of AD.

## Highlights

• The study identified 10 out of 17 polyphenols with favorable drug-likeness and lower toxicity, indicating their potential as therapeutic agents for Alzheimer's disease (AD).
• A range of in silico tools, including SwissADME, ProTox3.0, Swiss Target Prediction, and others, were used to establish the network pharmacology of polyphenols, revealing their multi-target potential in AD treatment.
• The study highlighted the mTOR pathway's role in AD and its impact through the autophagic mechanism, providing a deeper understanding of the disease's pathology and potential intervention points.
• The findings suggest that incorporating polyphenolic cocktails into the diet could enhance therapeutic outcomes against AD, offering a practical approach to disease management.
• The study calls for further research to explore additional pathways and genes to comprehensively understand the efficacy and mechanisms of polyphenols and their derivatives in preventing and treating AD.

## Graphical Abstract

**Figure:**
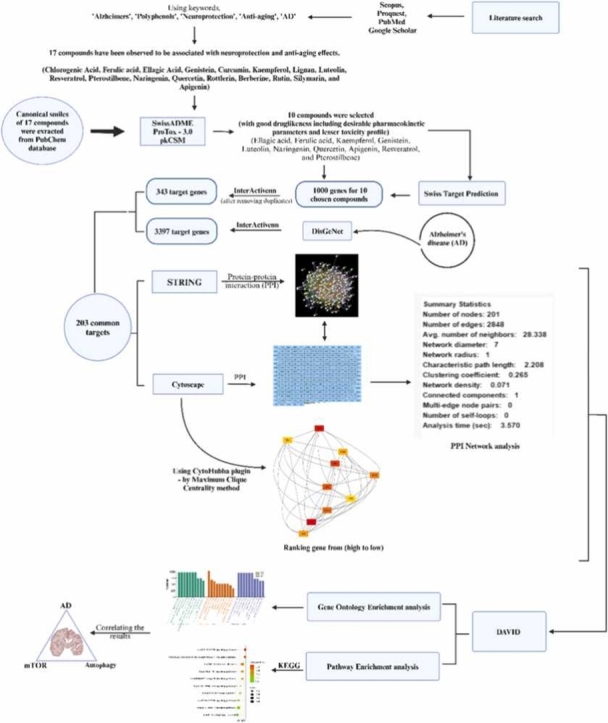

## 1 Introduction

Alzheimer’s disease (AD) primarily targets the brain leading to progressive cognitive decline and memory loss. The disease is characterized by the accumulation of abnormal protein aggregates, especially beta-amyloid plaques and tau tangles, that disrupt neuronal function and communication [1], [2]. The limited effectiveness of current pharmaceuticals has prompted the exploration of alternative therapies. One promising avenue is the use of natural polyphenols, abundant in fruits, vegetables, and plants, known for their potential neuroprotective effects against cognitive decline and AD pathogenesis [3]. Recent advances in bioinformatics and network pharmacology have enabled systematic exploration of the anti-AD properties of these compounds, establishing the complex interactions and therapeutic potentials of natural polyphenols against AD.

The last decade has witnessed accelerating interest in the prevention and treatment of neurodegenerative diseases, particularly AD, through natural polyphenols. This interest is rooted in the ability of polyphenols to modulate various biological pathways implicated in the onset and progression of AD [4]. Studies have highlighted how dietary polyphenols can exert neuroprotective [5] and pro-cognitive activities by modulating oxidative stress and inflammatory responses, which are cardinal features in AD pathophysiology [6]. Preclinical evidence supports the potential of polyphenols like resveratrol, curcumin, ellagic acid, quercetin, etc., in counteracting AD's pathogenic processes through their modulation of oxidative stress and inflammation [7].

Oxidative stress plays a pivotal role in AD's pathogenesis, serving as a target for therapeutic strategies. Natural polyphenolic compounds have been noted for their antioxidant neuroprotective effects and therapeutic potential for AD [8]. Also, compounds such as curcumin, epigallocatechin-3-gallate (EGCG), resveratrol, and tannic acid, have shown promising effects in decreasing amyloid-beta (Aβ) production, preventing or altering Aβ aggregation, and reducing oligomer cytotoxicity [9]. These natural molecules highlight the broad spectrum of polyphenols in offering potential AD therapeutic strategies.

The multifaceted actions of polyphenols offer significant therapeutic potential, addressing various pathological aspects of AD from amyloid-beta aggregation to oxidative stress and neuroinflammation [10]. Thus, natural polyphenols not only hold therapeutic promise but also inspire new research directions for developing novel AD treatments. Polyphenols are compounds with a wide spectrum of complex structures prevalent in diets based on plants. Polyphenols can be classified as either phenolic alcohols or phenolic acids. Based on the strength of their phenolic ring, polyphenols may be divided into many different forms, but the main groups include phenolic acids, flavonoids, stilbenes, phenolic alcohols, and lignans. Numerous edible and medicinal plants, as well as fruits, vegetables, tea, red wine, and extra virgin olive oil, are significant food sources that contain polyphenols [11]. These phytochemicals are known to shield human health from long-term degenerative diseases. They exhibit several protective properties against cancer, neurological disorders, atherosclerosis, stroke, and cardiovascular diseases [12], [13].

The integration of network pharmacology into allopathic medicine research has inaugurated a new era of drug discovery and development [14], characterized by a deeper understanding of drug actions at both the molecular and system levels [15]. This approach propels the study of drugs from a new perspective, emphasizing a holistic view over the reductionist methods traditionally employed in pharmaceutical research. It assesses the interplay between drugs and disease targets within the vast network of biological pathways, inherently relying on a synergistic approach to treatment.

## 2 Materials and method

### 2.1 Search strategy

Natural polyphenols have always been seen to exhibit beneficial effects on various diseases. Based on a literature survey that was carried out using various search engines such as Scopus, Proquest, PubMed, and Google Scholar, utilizing the keywords, ‘Alzheimers’, ‘polyphenols’ ‘neuroprotection’, ‘anti-aging’, and ‘AD’ [16], the following compounds including Chlorogenic Acid, Ferulic acid, Ellagic Acid, Genistein, Curcumin, Kaempferol, Lignan**,** Luteolin, Resveratrol, Pterostilbene, Naringenin, Quercetin, Rottlerin, Berberine, Rutin, Silymarin, and Apigenin were reflected as search results. These 17 compounds (refer to the structures of the ligands in the supplementary file) have been observed to be associated with neuroprotection and anti-aging effects and are beneficial against neurodegenerative diseases including Alzheimer’s disease [17], [18], [19], [20], [21], [22], [23], [24], [25], [26], [27], [28], [29], [30], [31], [32], [33].

### 2.2 Screening of compounds

#### 2.2.1 Drug-likeliness prediction

The canonical smiles of the selected 17 polyphenols (depicted in Table 1) were obtained from the PubChem database and entered into the SwissADME web server (http://www.swissadme.ch/) to assess their drug-likeliness. Several parameters were used to assess the drug-like properties of the polyphenols, including lead-likeness and bioavailability score, TPSA (Topological Polar Surface Area), which ascertain the brain permeability and the gastrointestinal absorption of the selected ligands [34], iLOGP (lipophilicity, solubility in lipid), ESOL Log S and ESOL class (water solubility), and the Lipinski rule of five (molecular mass should be less than 500 Dalton, lipophilicity should be high, hydrogen bond donors should be less than five, hydrogen bond acceptors should be less than ten, and molar refractivity within (40–130) [35]. A bioavailability value of F > 30 % is required, where F30 indicates a 30 % bioavailability. Bioavailability is the pace and degree of absorption of the active ingredients from a drug product into the bloodstream.

**Table 1:** List of compounds along with their canonical smiles used in our study.

**S.No**	**Compound Name**	**Canonical smiles**
1.	Chlorogenic Acid	C1C(C(C(CC1(C(=O)O)O)OC(=O)C=CC2=CC(=C(C=C2)O)O)O)O
2.	Ellagic Acid	C1=C2C3=C(C(=C1O)O)OC(=O)C4=CC(=C(C(=C43)OC2=O)O)O
3.	Curcumin	COC1=C(C=CC(=C1)C=CC(=O)CC(=O)C=CC2=CC(=C(C=C2)O)OC)O
4.	Ferulic acid	COC1=C(C=CC(=C1)C=CC(=O)O)O
5.	Kaempferol	C1=CC(=CC=C1C2=C(C(=O)C3=C(C=C(C=C3O2)O)O)O)O
6.	Genistein	C1=CC(=CC=C1C2=COC3=CC(=CC(=C3C2=O)O)O)O
7.	Lignan	CCOC(=O)C1C(C(=O)C2=CC(=C(C=C2C1C3=CC(=C(C(=C3)OC)OC)OC)OC)OC)C
8.	Luteolin	C1=CC(=C(C=C1C2=CC(=O)C3=C(C=C(C=C3O2)O)O)O)O
9.	Naringenin	C1C(OC2=CC(=CC(=C2C1=O)O)O)C3=CC=C(C=C3)O
10.	Quercetin	C1=CC(=C(C=C1C2=C(C(=O)C3=C(C=C(C=C3O2)O)O)O)O)O
11.	Resveratrol	C1=CC(=CC=C1C=CC2=CC(=CC(=C2)O)O)O
12.	Rottlerin	CC1=C(C(=C(C(=C1O)C(=O)C)O)CC2=C(C(=C3C(=C2O)C=CC(O3)(C)C)C(=O)C=CC4=CC=CC=C4)O)O
13.	Rutin	CC1C(C(C(C(O1)OCC2C(C(C(C(O2)OC3=C(OC4=CC(=CC(=C4C3=O)O)O)C5=CC(=C(C=C5)O)O)O)O)O)O)O)O
14.	Silymarin	COC1=C(C=CC(=C1)C2C(OC3=C(O2)C=C(C=C3)C4C(C(=O)C5=C(C=C(C=C5O4)O)O)O)CO)O
15.	Apigenin	C1=CC(=CC=C1C2=CC(=O)C3=C(C=C(C=C3O2)O)O)O
16.	Berberine	COC1=C(C2=C[N+]3=C(C=C2C=C1)C4=CC5=C(C=C4CC3)OCO5)OC
17.	Pterostilbene	COC1=CC(=CC(=C1)C=CC2=CC=C(C=C2)O)OC

With a Boiled Egg graphical interface, the drug-likeness is represented by ADME (Absorption, Distribution, Metabolism, and Excretion) characteristics, where the X and Y axes were displayed with TPSA and WLogP values, respectively. TPSA and WLogP stand for the ligand’s accessibility and lipophilicity, respectively.

#### 2.2.2 ADMET profiling

Utilizing the ProTox 3.0 web server (https://tox.charite.de/protox3/#), the toxicity profile of each of the 17 ligands was assessed[36]. The SMILES of the corresponding ligands were incorporated into the webserver to analyze and make an assumption of the compounds' LD_50_ value. The compounds were categorized into Class 1–6 based on their toxicity nature. Class 1 indicates the highly toxic nature of the compound and Class 6 indicates the non-toxic nature of the compound. A limit of Class 4 was chosen as the main filter to eliminate some compounds out of 17 compounds used in our study [37]. Additionally, pkCSM (https://biosig.unimelb.edu.au/pkcsm//) webserver was employed to extensively assess the comprehensive pharmacokinetic profile of the ligands including parameters such as water solubility, Caco2 permeability, Intestinal absorption, Skin permeability, P-glycoprotein inhibition, volume of distribution, BBB permeability, CNS permeability, Enzyme interactions, and Renal clearance were assessed extensively to filter the potential compounds against AD [38], [39].

#### 2.2.3 Prediction of putative targets

Following screening via the SwissADME, ProTox-3.0 databases, and pkCSM web server, the canonical SMILES of the 10 chosen compounds were entered into the Swiss target prediction website and then analyzed to anticipate the possible targets.

### 2.3 Protein-protein interaction (PPI) network construction

The great diversity, flexibility, and selectivity of protein-protein interactions (PPI) make them extremely important [40]. The functional relationships between major targets were found using the Search Tool for the Retrieval of Interacting Genes/Proteins (STRING) [41]. The CytoHubba plugin for Cytoscape was applied to the PPI network that was acquired from STRING to examine its core regulatory genes and identify relevant targets. Also, extended PPI network analysis was done to assess the clustering coefficient, number of nodes, number of edges, network radius, and average number of neighbours to validate the PPI [42].

### 2.4 Gene Ontology and KEGG pathway enrichment analysis

The Kyoto Encyclopedia of Genes and Genomes (KEGG) pathway [43] enrichment and Gene Ontology (GO) enrichment were performed by employing the Database for Annotation, Visualization, and Integrated Discovery (DAVID) to identify the interactions between molecules and biological processes linked to specific hub genes.

### 2.5 Statistical analyses

Statistical techniques such as network centrality measures, and pathway analysis were performed to evaluate the significance of our findings.

## 3 Results

### 3.1 Screening of compounds

#### 3.1.1 Drug-likeliness prediction

**Fig. 1:**
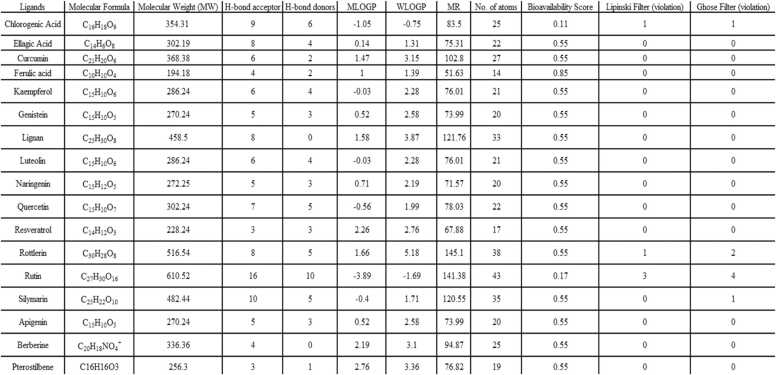
Drug-likeness Prediction using the SwissADME database. Lipinski filter: MW≤500, MLOGP ≤4.15, N or O ≤10, NH or OH≤5; Ghosh filter:160≤MW≤480, −0.4≤WLOGP≤5.6, 40 ≤MR ≤130, 20 ≤atoms ≤70, F ≥ 30 %.

Using Swiss ADME, the ligands were predicted for drug-likeness by utilizing the Ghosh filter and Lipinski's rule of five. For every ligand, drug-likeness properties such as logP, MW, number of hydrogen-bond donors (HBD), number of hydrogen-bond acceptors (HBA), number of hydrogen-bond donors (HBD), MLOGP, WLOGP, MR, and number of atoms were predicted. Out of 17 ligands, 13 compounds exhibited drug-likeness characteristics after passing the Lipinski and Ghose Filter with no violations. The compounds such as Chlorogenic acid, Rottlerin, Rutin, and Silymarin showed violations against Lipinski and Ghose filters. Fig. 1 depicts the findings for each ligand's drug-likeness.

**Fig. 2:**
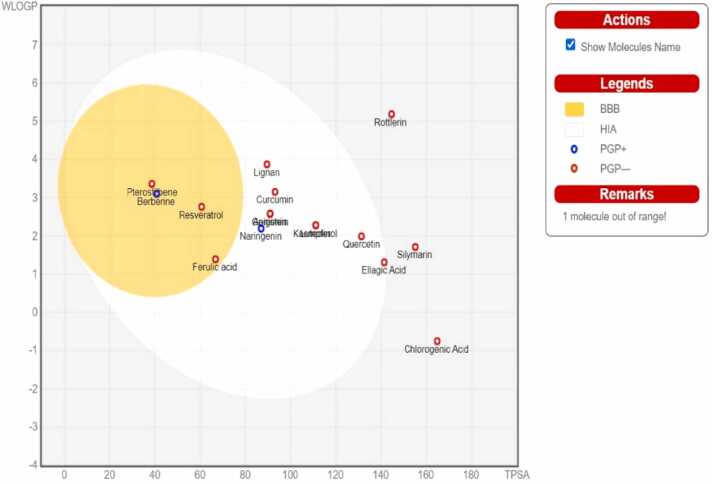
Boiled egg graphical representation of 17 polyphenols using SwissADME.

The boiled egg graphical representation (displayed in Fig. 2), showed that Pterostilbene, Berberine, Resveratrol, and Ferulic acid have a good probability of crossing the BBB and the other polyphenols have a lower probability of crossing BBB, except Chlorogenic acid, Silymarin, and Rottlerin which were found to be non-brain penetrant [44], [45].

#### 3.1.2 ADMET profiling

**Fig. 3:**
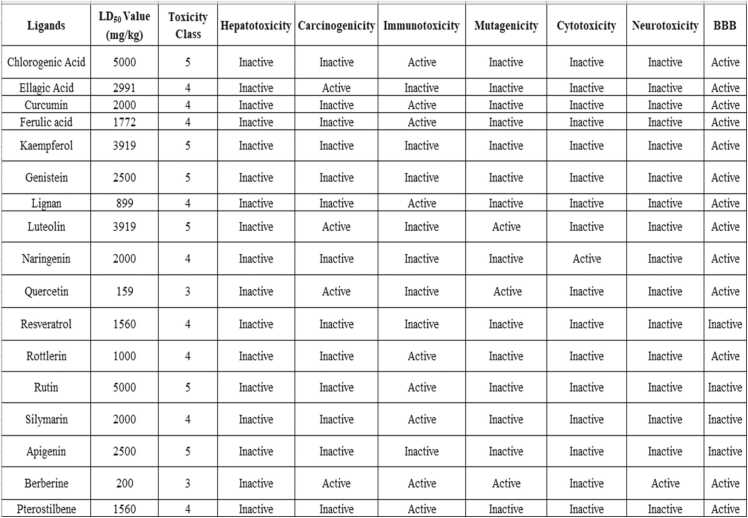
Toxicity results using ProTox – 3.0 Software. LD_50_: Median Lethal Dose; BBB: Blood Brain Barrier.

Utilizing the ProTox 3.0 web server toxicity profile for all 17 polyphenols was assessed (depicted in Fig. 3). The results indicated that except for lignan, berberine, and quercetin, the remaining ligands showed high LD_50_ values. The BBB permeability nature of the compounds predicted using ProTox – 3.0 was found to be inconsistent with the results evaluated through the SwissADME Boiled egg plot. To minimize this bias, the pkCSM web server was used to extensively study the comprehensive ADMET (absorption, distribution, metabolism, excretion, and toxicity) properties of the compounds

Generally, in the pkCSM web server, the value of water solubility ranges from −6 to 0. Lower values indicate poorer water solubility which may have challenges in absorption and formulation. The range of Caco2 permeability between −1 and 1.5 implies the ability of the compound to permeate the intestinal lining. A higher value suggests good permeability and potential for high oral bioavailability, while a lower value indicates poor absorption. The range of intestinal absorption lies between 0 and 100. Compounds with values above 80 % are considered to have high absorption.

The skin permeability value ranges between −3 and −1. Lower values suggest low skin permeability of the compounds indicating the compound’s relevancy for topical or transdermal drug delivery. The P-glycoprotein inhibition property indicates the compound potentially affects the drug efflux and increases the intracellular drug concentration. The volume of distribution value ranges between −2 and 2. A higher value indicates the wide distribution of the compound throughout the tissues, and a lower value indicates the confinement of the compound to the plasma.

BBB permeability value ranges between −3 and 1. Values closer to 0 or positive indicate good BBB permeability. Also, the CNS permeability ranges between −4 and 0. Higher values indicate better CNS penetration, which is very important for drugs targeting the brain. The enzyme interaction property indicates the possibility of the compound causing drug-drug interactions. Total clearance value ranges between −1 and 2. Higher values suggest faster clearance, which can affect dosing frequency and duration of action. The substrate property of compounds for Renal Organic cation transporter 2 implies the renal excretion via this transporter. If the compounds show no interaction, it indicates alternative pathways for renal excretion.

**Fig. 4:**
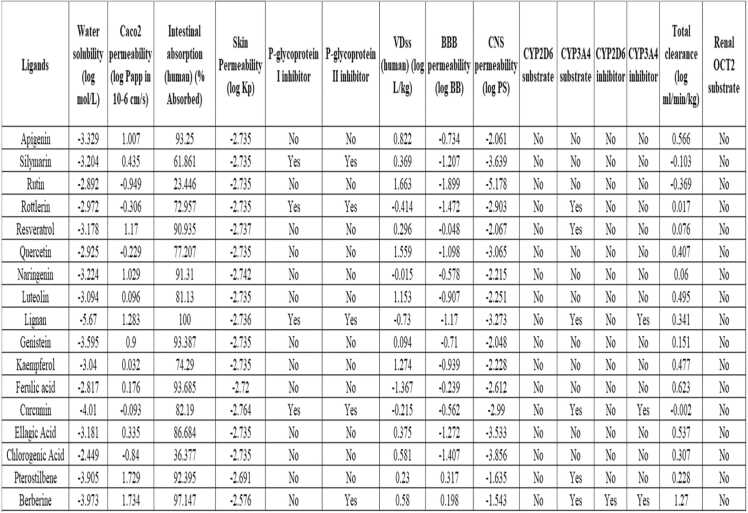
Comprehensive ADMET Profiling using pkCSM software. Caco2: Cancer coli-2 cells; VD_ss_: The steady-state Volume of distribution; CNS permeability: Central Nervous System permeability; CYP2D6: Cytochrome P450 2D6; CYP3A4: Cytochrome P450 3A4; Renal OCT2: Renal Organic cation transporter 2.

From the pkCSM webserver analysis (depicted in Fig. 4), it was found that 10 compounds including Ellagic acid, Ferulic acid, Kaempferol, Genistein, Luteolin, Naringenin, Quercetin, Apigenin, Resveratrol, and Pterostilbene exerted moderate to good oral bioavailability with variations depending on solubility and glycoside forms, their ability to cross BBB. Also, they have shown extensive phase II metabolism (especially glucuronidation and sulfation) and efficient renal clearance primarily through urine as conjugated metabolites. These properties play a crucial role in determining their potential therapeutic applications and dosage forms, so they were selected for further study, and 7 ligands such as Chlorogenic acid, Curcumin, Lignan, Rottlerin, Rutin, Silymarin, and Berberine with unfavourable pharmacokinetic profiles were removed from the further study.

#### 3.1.3 Prediction of putative targets

**Fig. 5:**
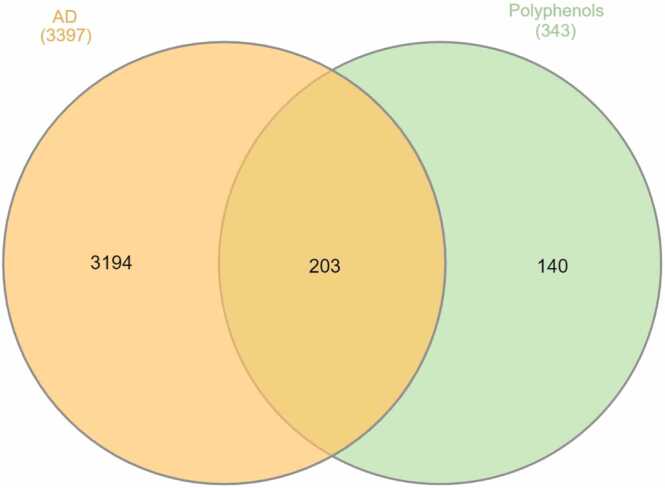
Venn diagram showing the common genes between AD and the selected polyphenols.

Using the Swiss Target Prediction database, a total of 1000 target genes (refer to the supplementary file) were identified. After discarding the duplicated genes in the InterActiVenn (http://www.interactivenn.net/), 343 putative target genes of 10 chosen active compounds were obtained (refer to the supplementary file). Following the identification of drugs' promising targets, 3397 genes linked to AD were obtained from the DisGeNET database (refer to the supplementary file). Afterward, a Venn diagram was used to anticipate the shared targets of the compound-related genes and AD (shown in Fig. 5). A total of 203 putative genes that protect against AD were chosen and regarded as hub targets.

### 3.2 Protein-protein interaction (PPI) network construction

**Fig. 6:**
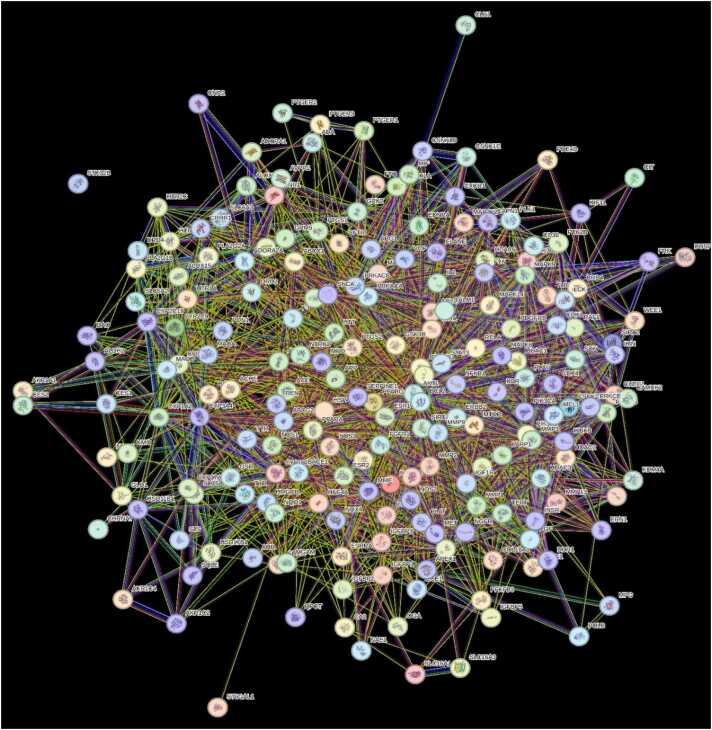
PPI Network analysis between selected polyphenols and targets for AD obtained using STRING Database (12.0).

**Fig. 7:**
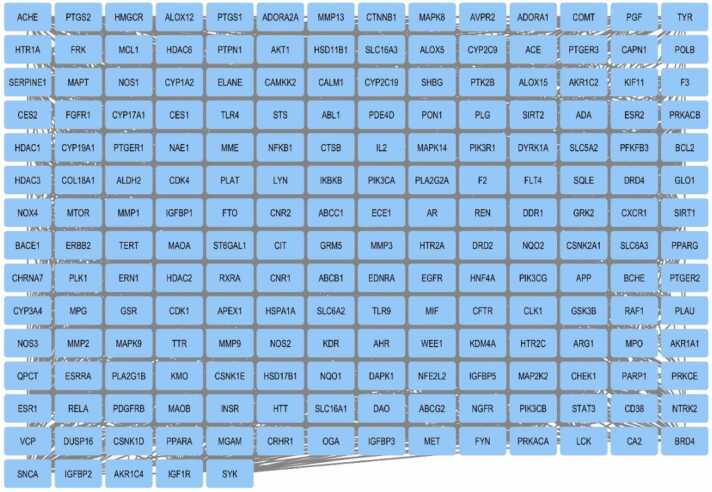
PPI Network using Cytoscape (v3.10.1).

**Fig. 8:**
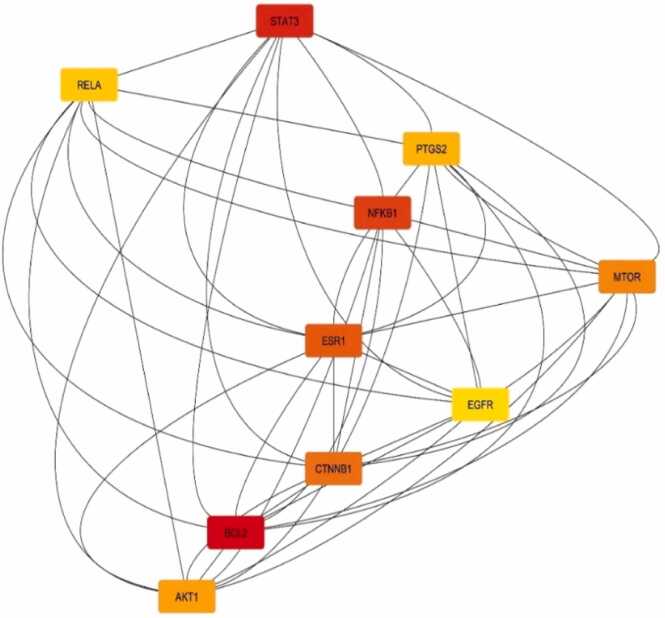
Top 10 genes in Cytoscape (v3.10.1) sorted by MCC method. The node colour changes from red to yellow reflecting the rank from high to low in the network.

**Fig. 9:**
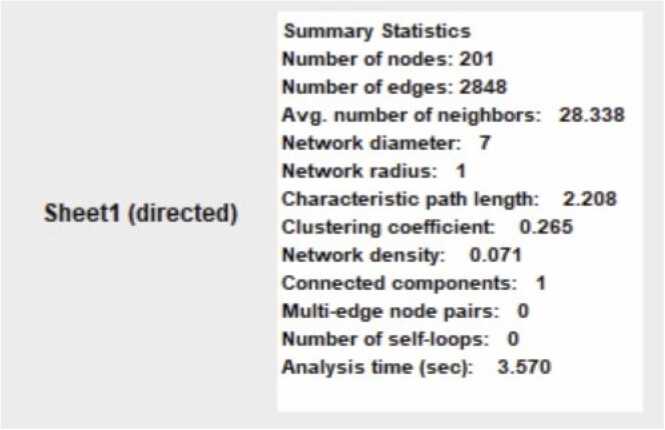
Metrics of PPI network analysis performed using Cytoscape.

One useful resource for predicting protein-protein interactions is the STRING (Search Tool for the Retrieval of Interacting Genes) database. The PPI network (displayed in Fig. 6) of gene lists was predicted using the STRING database version 12.0 (refer to the supplementary file). Cytoscape (v3.10.1) was used to show the predicted PPI network (shown in Fig. 7). Using Maximal Clique Centrality (MCC) topological analysis, the Cytoscape plugin CytoHubba (depicted in Fig. 8) was able to identify the top 10 core genes (BCL2, NFKB1, STAT3, ESR1, CTNNB1, MTOR, AKT1, PTGS2, RELA, EGFR). Also, detailed protein-protein interaction network analysis was performed and it indicated that there were 201 proteins (nodes) in the network (displayed in Fig. 9). About 2848 interactions were found between the proteins with an average of each protein interacting with approximately 28 other proteins with a network diameter of 7 steps and radius of 1 suggesting the network is highly connected. This network centrality measure showed significant validation of PPI performed in our study.

### 3.3 GO (Gene Ontology) enrichment analysis

**Fig. 10:**
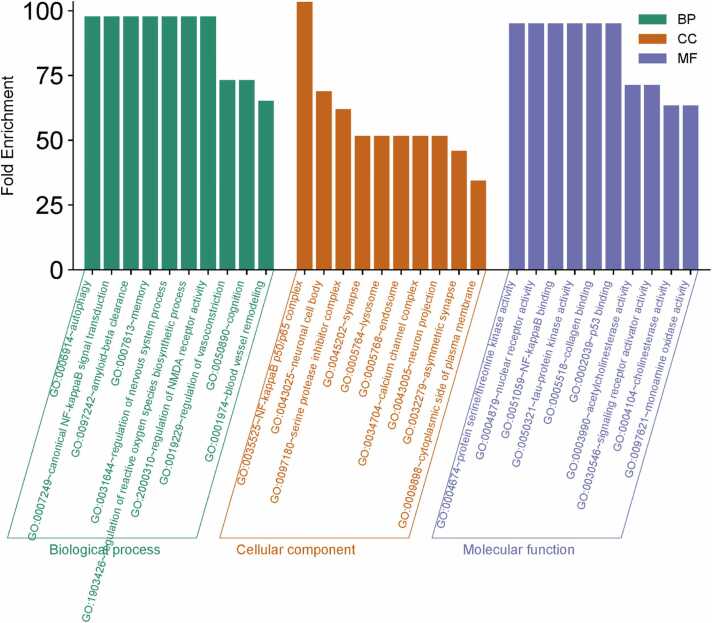
Target protein GO enrichment analysis. The quantity of GO entries (P < 0.05) in the functional categories of biological process (BP), molecular function (MF), and cell composition (CC).

Functional annotation and enrichment analysis were carried out using the Database for Annotation, Visualization, and Integrated Discovery (DAVID) [46]. DAVID was used to estimate the function of the primary targets at three different levels: cellular component (CC), molecular function (MF), and biological process (BP). To further investigate the 203 selected target genes, GO enrichment analysis was executed on them (refer to the supplementary file). GO enrichment analysis revealed that most of the target genes were involved in biological processes (BP), cellular components (CC), and molecular functions (MF) (depicted in Fig. 10). Autophagy, Nuclear factor kappa B transduction, Amyloid beta clearance, memory process, and regulation of ROS biosynthetic process in particular dominated the enriched BP ontologies. In the CC analysis, the amyloid and microsome account for the majority (203 target genes). Tyrosine-protein kinase, dioxygenase, peroxidase, and other enzymes dominated the enriched MF ontologies.

### 3.4 KEGG (Kyoto Encyclopedia of Genes and Genomes) enrichment analysis

**Fig. 11:**
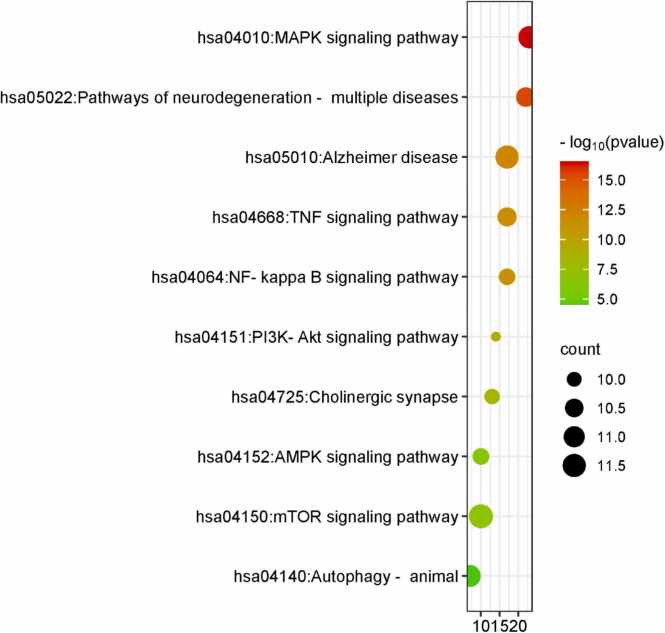
Top 10 KEGG terms of hub genes.

Following the GO enrichment analysis, KEGG pathway enrichment analysis of the 203 targeted genes was performed using the DAVID database (refer to the supplementary file). The results of the KEGG pathway enrichment analysis showed that 277 signal pathways and 203 putative target genes had significant associations (FDR < 0.05). In Fig. 11, the top 10 pathways with the highest enrichment ratios are shown.

According to the KEGG enrichment analysis of the top 10 pathways associated between the selected polyphenols and AD, the most relevant pathway is the mTOR Signaling Pathway involving the hub genes. Now previously using Maximal Clique Centrality (MCC) topological analysis, Cytoscape plugin CytoHubba we found the top 10 target genes involved between the disease and the drug [47]. By correlating these results, we can conclude that the mTOR signaling pathway can play a vital role in AD [48]. Therefore, this can be focused on treating AD using the selected polyphenols to get a more appropriate outcome.

## 4 Discussion

The study of natural polyphenols in the context of AD using bioinformatic tools presents a promising frontier in neurodegenerative disease research. Advancements in bioinformatics have facilitated the identification of targets, biomarkers, pathways, and potential therapeutics, integrating computational methods into the study of complex diseases like AD [14]. This approach enhances the understanding of AD's pathological mechanisms and the draggability of molecular targets, leveraging the vast potential of natural polyphenols systematically and efficiently.

This work serves as both a benchmark for the preliminary screening of some polyphenols and a novel therapeutic idea for more investigation into the processes underlying the use of polyphenols in the treatment of AD. After the initial scrutiny, 10 compounds with desirable pharmacokinetic parameters were further subjected to PPI and Enrichment analysis. The anti-AD targets of certain polyphenols were mostly related to the autophagy mechanism, memory, and regulation of the ROS biosynthetic process as per GO functional analysis. Also, the studies on the KEGG pathway enrichment analysis showed that targets were linked to pathways relevant to AD. The network topological analysis also revealed several high-degree nodes, suggesting key genes that may play critical roles in Alzheimer's disease pathology. Modules identified within the network correspond to known AD pathways, such as neuroinflammation and amyloid processing, and the network is highly connected. This high level of connectivity often enhances the network's functionality and resilience.

**Fig. 12:**
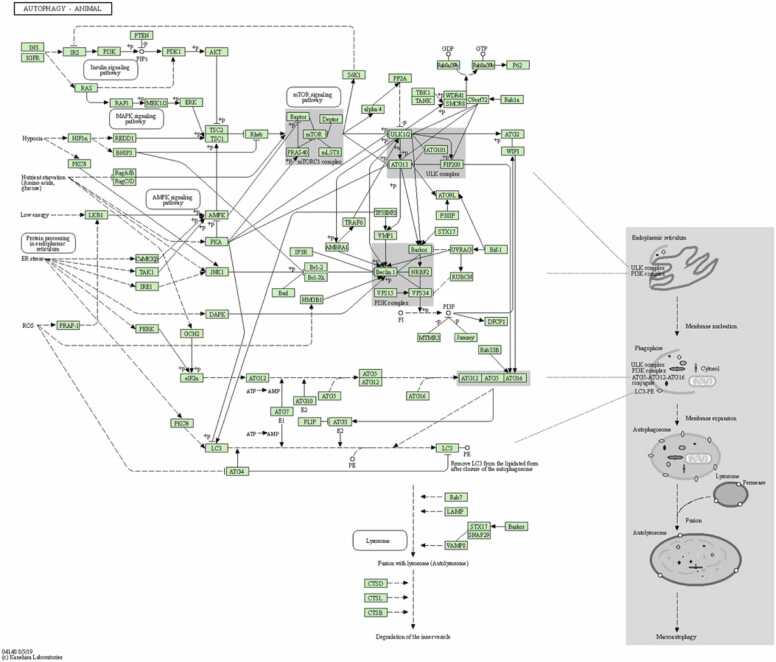
Autophagy pathway (KEGG Id: hsa04140), highlighting its association with the mTOR signaling pathway (KEGG map)[43].

This current research supports the existing interconnection between the mTOR signaling pathway, the Autophagy mechanism, and AD [49]. An intricate network of molecular signal transduction pathways interacts in the pathogenic process of AD. Autophagy is a catabolic process by which cells degrade and recycle their components. It involves the formation of double-membrane vesicles called autophagosomes that engulf cytoplasmic material, including damaged organelles and proteins. These autophagosomes fuse with lysosomes to form autolysosomes, where the contents are degraded and recycled [50]. The pathophysiology of AD involves upregulation of the mTOR signaling pathway, which plays an essential role in Autophagy regulation and also other associated mechanisms (depicted in Fig. 12). The mTOR pathway negatively regulates autophagy [51]. Impaired autophagy is linked to the accumulation of protein aggregates in diseases like AD. Modulating mTOR and autophagy may offer therapeutic benefits against the disease. The mTOR signaling system and autophagy are inextricably linked.

A growing body of scientific evidence suggests that polyphenols usually have a neuroprotective effect on AD pathology. Some of them usually act by the anti-oxidant potential to combat the free radicals which in turn results in the inhibition of apoptosis and also helps in regulating the autophagy mechanism [52], [53]. The current study meticulously explains the association between certain polyphenols and the mTOR signaling pathway and the etiology of AD, offering an analytical framework for the use of polyphenols for the management of AD [54], [55]. Still, several other pathways need to be investigated in future studies.

## 5 Conclusion

Alzheimer’s disease has substantial financial implications and is the second most common cause of death and disability globally. Despite the vast amount of research exploring multiple paths leading to AD and increasing awareness of the pathogenesis of the disease, the difficulty of translating research into clinical settings has significantly hampered developments in AD research. Our study has shed light on the possible interaction between certain polyphenols and the mTOR pathway, through which they can influence the regulation of the Autophagy mechanism, providing betterment against AD. Compared to single natural or pharmaceutical chemical substances, significantly more effective outcomes can be acquired in a shorter amount of time by ingesting foods or natural items with an increased and diverse level of polyphenols (polyphenol cocktail). Therefore, further research is needed in the future to explore other pathways and genes to provide in-depth knowledge in the prevention of AD and also laboratory experiments are warranted to further explore the pharmacological potential of the shortlisted polyphenolic compounds and their derivatives with a focus on developing a promising drug candidate against AD.

## CRediT authorship contribution statement

**Arunkumar Subramanian:** Writing – original draft, Project administration, Investigation, Data curation, Conceptualization. **T. Tamilanban:** Writing – review & editing, Validation, Project administration, Investigation, Data curation, Conceptualization. **Vetriselvan Subramaniyan:** Writing – original draft, Validation, Investigation, Data curation, Conceptualization. **Mahendran Sekar:** Writing – review & editing, Project administration, Investigation, Conceptualization. **Vipin Kumar:** Writing – original draft, Visualization, Project administration, Investigation, Conceptualization. **Ashok Kumar Janakiraman:** Writing – review & editing, Project administration, Investigation. **Saminathan Kayarohanam:** Writing – review & editing, Project administration, Investigation.

## Declaration of Competing Interest

The authors declare that they have no known competing financial interests or personal relationships that could have appeared to influence the work reported in this paper.

## Data Availability

The authors are unable or have chosen not to specify which data has been used.

## References

[1] Scheltens P., Blennow K., Breteler M.M.B., de Strooper B., Frisoni G.B., Salloway S., Van der Flier W.M. (2016). Alzheimer’s disease. Lancet.

[2] Alzheimers disease: a brief review, J. Exp. Neurol., 12020, 10.33696/Neurol.1.015..

[3] Albadrani H.M., Chauhan P., Ashique S., Babu M.A., Iqbal D., Almutary A.G., Abomughaid M.M., Kamal M., Paiva-Santos A.C., Alsaweed M., Hamed M., Sachdeva P., Dewanjee S., Jha S.K., Ojha S., Slama P., Jha N.K. (2024). Mechanistic insights into the potential role of dietary polyphenols and their nanoformulation in the management of Alzheimer’s disease. Biomed. Pharmacother..

[4] El Gaamouch F., Chen F., Ho L., Lin H.-Y., Yuan C., Wong J., Wang J. (2022). Benefits of dietary polyphenols in Alzheimer’s disease. Front. Aging Neurosci..

[5] Subramanian A., T. T, Kumarasamy V., Sekar M., Subramaniyan V., Wong L.S. (2024). Design, synthesis and invitro pharmacological evaluation of novel resveratrol surrogate molecules against Alzheimer’s disease. Chem. Biodivers..

[6] Colizzi C. (2019). The protective effects of polyphenols on Alzheimer’s disease: a systematic review. Alzheimers Dement..

[7] Subramanian A., Tamilanban T., Sekar M., Begum M.Y., Atiya A., Ramachawolran G., Wong L.S., Subramaniyan V., Gan S.H., Mat Rani N.N.I., Wu Y.S., Chinni S.V., Fuloria S., Fuloria N.K. (2023). Neuroprotective potential of Marsilea quadrifolia Linn against monosodium glutamate-induced excitotoxicity in rats. Front. Pharmacol..

[8] Choi D.-Y., Lee Y.-J., Hong J.T., Lee H.-J. (2012). Antioxidant properties of natural polyphenols and their therapeutic potentials for Alzheimer’s disease. Brain Res. Bull..

[9] Phan H.T.T., Samarat K., Takamura Y., Azo-Oussou A.F., Nakazono Y., Vestergaard M.C. (2019). Polyphenols modulate alzheimer’s amyloid beta aggregation in a structure-dependent manner. Nutrients.

[10] Grabska-Kobyłecka I., Szpakowski P., Król A., Książek-Winiarek D., Kobyłecki A., Głąbiński A., Nowak D. (2023). Polyphenols and their impact on the prevention of neurodegenerative diseases and development. Nutrients.

[11] Pandey K.B., Rizvi S.I. (2009). Plant polyphenols as dietary antioxidants in human health and disease. Oxid. Med. Cell. Longev..

[12] Bukhari S.N.A. (2022). Dietary polyphenols as therapeutic intervention for Alzheimer’s disease: a mechanistic insight. Antioxidants.

[13] Tresserra-Rimbau A., Arranz S., Vallverdu-Queralt A. (2017). New insights into the benefits of polyphenols in chronic diseases. Oxid. Med. Cell. Longev..

[14] Singh S.K., Kumar A., Singh R.B., Ghosh P., Bajad N.G. (2022). Recent APplications of Bioinformatics in Target Identification and Drug Discovery for Alzheimer’s disease. Curr. Top. Med. Chem..

[15] Chandran U., Mehendale N., Patil S., Chaguturu R., Patwardhan B. (2017). Innovative Approaches in Drug Discovery.

[16] Chigbu U.E., Atiku S.O., Du Plessis C.C. (2023). The science of literature reviews: searching, identifying, selecting, and synthesising. Publications.

[17] Gao L., Li X., Meng S., Ma T., Wan L., Xu S. (2020). Chlorogenic Acid Alleviates Aβ25-35-induced autophagy and cognitive impairment via the mTOR/TFEB signaling pathway. Drug Des. Dev. Ther..

[18] Gupta A., Singh A.K., Kumar R., Jamieson S., Pandey A.K., Bishayee A. (2021). Neuroprotective potential of ellagic acid: a critical review. Adv. Nutr..

[19] Mishra S., Palanivelu K. (2008). The effect of curcumin (turmeric) on Alzheimer’s disease: an overview. Ann. Indian Acad. Neurol..

[20] Meng G., Meng X., Ma X., Zhang G., Hu X., Jin A., Zhao Y., Liu X. (2018). Application of ferulic acid for Alzheimer’s disease: combination of text mining and experimental validation. Front. Neuroinform..

[21] Dong X., Zhou S., Nao J. (2023). Kaempferol as a therapeutic agent in Alzheimer’s disease: evidence from preclinical studies. Ageing Res. Rev..

[22] Uddin Md.S., Kabir Md.T. (2019). Emerging signal regulating potential of genistein against Alzheimer’s disease: a promising molecule of interest. Front. Cell. Dev. Biol..

[23] Han N., Wen Y., Liu Z., Zhai J., Li S., Yin J. (2022). Advances in the roles and mechanisms of lignans against Alzheimer’s disease. Front. Pharmacol..

[24] He Z., Li X., Wang Z., Cao Y., Han S., Li N., Cai J., Cheng S., Liu Q. (2023). Protective effects of luteolin against amyloid beta-induced oxidative stress and mitochondrial impairments through peroxisome proliferator-activated receptor γ-dependent mechanism in Alzheimer’s disease. Redox Biol..

[25] Poudineh S., Poudineh M., Ghotbi T., Azizi F., Karami N., Zolfaghari Z., Gheisari F., Hormozi M. (2022). Neuropharmaceutical properties of naringin against Alzheimer’s and Parkinson’s diseases. Galen. Med. J..

[26] Khan H., Ullah H., Aschner M., Cheang W.S., Akkol E.K. (2019). Neuroprotective Effects of Quercetin in Alzheimer’s disease. Biomolecules.

[27] Gomes B.A.Q., Silva J.P.B., Romeiro C.F.R., dos Santos S.M., Rodrigues C.A., Gonçalves P.R., Sakai J.T., Mendes P.F.S., Varela E.L.P., Monteiro M.C. (2018). Neuroprotective mechanisms of resveratrol in Alzheimer’s disease: role of SIRT1. Oxid. Med. Cell. Longev..

[28] Siposova K., Kozar T., Huntosova V., Tomkova S., Musatov A. (2019). Inhibition of amyloid fibril formation and disassembly of pre-formed fibrils by natural polyphenol rottlerin. Biochim. Et. Biophys. Acta (BBA) - Proteins Proteom..

[29] Xu P., Wang S., Yu X., Su Y., Wang T., Zhou W., Zhang H., Wang Y., Liu R. (2014). Rutin improves spatial memory in Alzheimer’s disease transgenic mice by reducing Aβ oligomer level and attenuating oxidative stress and neuroinflammation. Behav. Brain Res..

[30] Guo H., Cao H., Cui X., Zheng W., Wang S., Yu J., Chen Z. (2019). Silymarin’s inhibition and treatment effects for Alzheimer’s disease. Molecules.

[31] Zhao L., Wang J.-L., Liu R., Li X.-X., Li J.-F., Zhang L. (2013). Neuroprotective, anti-amyloidogenic and neurotrophic effects of apigenin in an Alzheimer’s disease mouse model. Molecules.

[32] Cai Z., Wang C., Yang W. (2016). Role of berberine in Alzheimer’s disease. Neuropsychiatr. Dis. Treat..

[33] Zhu L., Lu F., Zhang X., Liu S., Mu P. (2022). SIRT1 is involved in the neuroprotection of pterostilbene against amyloid β 25–35-induced cognitive deficits in mice. Front. Pharmacol..

[34] Ertl P., Rohde B., Selzer P. (2000). Fast calculation of molecular polar surface area as a sum of fragment-based contributions and its application to the prediction of drug transport properties. J. Med. Chem..

[35] Lipinski C.A. (2004). Lead- and drug-like compounds: the rule-of-five revolution. Drug Discov. Today Technol..

[36] Banerjee P., Eckert A.O., Schrey A.K., Preissner R. (2018). ProTox-II: a webserver for the prediction of toxicity of chemicals. Nucleic Acids Res..

[37] Banerjee P., Kemmler E., Dunkel M., Preissner R. (2024). ProTox 3.0: a webserver for the prediction of toxicity of chemicals. Nucleic Acids Res..

[38] Krüger A., Gonçalves Maltarollo V., Wrenger C., Kronenberger T. (2020). Drug Discovery and Development - New Advances.

[39] Pires D.E.V., Blundell T.L., Ascher D.B. (2015). pkCSM: predicting small-molecule pharmacokinetic and toxicity properties using graph-based signatures. J. Med. Chem..

[40] Noor F., Saleem M.H., Javed M.R., Chen J.-T., Ashfaq U.A., Okla M.K., Abdel-Maksoud M.A., Alwasel Y.A., Al-Qahtani W.H., Alshaya H., Yasin G., Aslam S. (2022). Comprehensive computational analysis reveals H5N1 influenza virus-encoded miRNAs and host-specific targets associated with antiviral immune responses and protein binding. PLoS One.

[41] Mering C. v (2003). STRING: a database of predicted functional associations between proteins. Nucleic Acids Res..

[42] Koh G.C.K.W., Porras P., Aranda B., Hermjakob H., Orchard S.E. (2012). Analyzing protein–protein interaction networks. J. Proteome Res..

[43] Kanehisa M. (2000). KEGG: kyoto encyclopedia of genes and genomes. Nucleic Acids Res..

[44] Daina A., Zoete V. (2016). A BOILED-Egg to predict gastrointestinal absorption and brain penetration of small molecules. ChemMedChem.

[45] Daina A., Michielin O., Zoete V. (2017). SwissADME: a free web tool to evaluate pharmacokinetics, drug-likeness and medicinal chemistry friendliness of small molecules. Sci. Rep..

[46] Huang D., Sherman B.T., Tan Q., Collins J.R., Alvord W.G., Roayaei J., Stephens R., Baseler M.W., Lane H.C., Lempicki R.A. (2007). The DAVID Gene Functional Classification Tool: a novel biological module-centric algorithm to functionally analyze large gene lists. Genome Biol..

[47] Chin C.-H., Chen S.-H., Wu H.-H., Ho C.-W., Ko M.-T., Lin C.-Y. (2014). cytoHubba: identifying hub objects and sub-networks from complex interactome. BMC Syst. Biol..

[48] Subramanian A., Tamilanban T., Alsayari A., Ramachawolran G., Wong L.S., Sekar M., Gan S.H., Subramaniyan V., Chinni S.V., Izzati Mat Rani N.N., Suryadevara N., Wahab S. (2022). Trilateral association of autophagy, mTOR and Alzheimer’s disease: Potential pathway in the development for Alzheimer’s disease therapy. Front Pharmacol..

[49] Querfurth H., Lee H.-K. (2021). Mammalian/mechanistic target of rapamycin (mTOR) complexes in neurodegeneration. Mol. Neurodegener..

[50] Glick D., Barth S., Macleod K.F. (2010). Autophagy: cellular and molecular mechanisms. J. Pathol..

[51] Cai Z., Zhou Y., Xiao M., Yan L.-J., He W. (2015). Activation of mTOR: a culprit of Alzheimer’s disease?. Neuropsychiatr. Dis. Treat..

[52] Aman Y., Schmauck-Medina T., Hansen M., Morimoto R.I., Simon A.K., Bjedov I., Palikaras K., Simonsen A., Johansen T., Tavernarakis N., Rubinsztein D.C., Partridge L., Kroemer G., Labbadia J., Fang E.F. (2021). Autophagy in healthy aging and disease. Nat. Aging.

[53] Klionsky D.J. (2008). Autophagy revisited: a conversation with Christian de Duve. Autophagy.

[54] Liu Q., Jin Z., Xu Z., Yang H., Li L., Li G., Li F., Gu S., Zong S., Zhou J., Cao L., Wang Z., Xiao W. (2019). Antioxidant effects of ginkgolides and bilobalide against cerebral ischemia injury by activating the Akt/Nrf2 pathway in vitro and in vivo. Cell Stress Chaperon.

[55] Metcalf D.J., García-Arencibia M., Hochfeld W.E., Rubinsztein D.C. (2012). Autophagy and misfolded proteins in neurodegeneration. Exp. Neurol..

