# Supplementary material for: Establishing network pharmacology between natural polyphenols and Alzheimer’s disease using bioinformatic tools – An advancement in Alzheimer’s research
Source: Toxicol Rep. 2024 Aug 23;13:101715. doi: 10.1016/j.toxrep.2024.101715 (PMC11402327; doi:10.1016/j.toxrep.2024.101715)
Supplement: Supplementary file 1 — Supplementary material [file mmc1.docx]

**Establishing Network Pharmacology between Natural Polyphenols and Alzheimer’s Disease using Bioinformatic Tools – An Advancement in Alzheimer’s Research**

Arunkumar Subramanian^1^, Tamilanban T^1^***,** Vetriselvan Subramaniyan^2^*, Mahendran Sekar^3^, Vipin Kumar^4^, Ashok Kumar Janakiraman^5^, Saminathan Kayarohanam^6^

^1^Department of Pharmacology, SRM College of Pharmacy, SRM Institute of Science and Technology, Kattankulathur, Chengalpattu, Tamilnadu – 603203, India.

^2^Department of Medical Sciences, School of Medical and Life Sciences, Sunway University Jalan University, Bandar Sunway 47500 Selangor Darul Ehsan, Malaysia

^3^School of Pharmacy, Monash University Malaysia, Bandar Sunway, Subang Jaya 47500, Selangor, Malaysia.

^4^Department of Pharmaceutical Sciences, Gurukul Kangri (Deemed to be University) Haridwar, 249404 India.

^5^Faculty of Pharmaceutical Sciences, UCSI University, 56000 Cheras, Kuala Lumpur, Malaysia.

^6^Faculty of Bioeconomics and Health Sciences, University Geomatika Malaysia, Kuala Lumpur 54200, Malaysia.

*Correspondence: Tamilanban T ([tamilant@srmist.edu.in](mailto:tamilant@srmist.edu.in)); Vetriselvan Subramaniyan ([subramaniyan.vetriselvan@monash.edu](mailto:subramaniyan.vetriselvan@monash.edu))

Contents

1. File 1: Table (a) depicting List of 17 Compounds used in the study and their structures
2. File 2 showing detailed toxicity results predicted using ProTox – 3.0 software
3. File 3 depicting targets for selected polyphenols
4. File 4 displaying the list of target genes for the disease
5. File 5 depicting the work flow in InterActiVenn software
6. File 6 displaying PPI using STRING database
7. File 7 showing PPI network analysis using Cytoscape
8. File 8 depicting the ranking of genes CytoHubba plug-in
9. File 9 showing workflow of data used for GO enrichment analysis
10. File 10 depicting workflow of data used for Pathway enrichment analysis
