# Supplementary material for: Establishing network pharmacology between natural polyphenols and Alzheimer’s disease using bioinformatic tools – An advancement in Alzheimer’s research
Source: Toxicol Rep. 2024 Aug 23;13:101715. doi: 10.1016/j.toxrep.2024.101715 (PMC11402327; doi:10.1016/j.toxrep.2024.101715)
Supplement: Supplementary file 4 — Supplementary material [file mmc2.docx]

**Table (a): List of 17 Compounds used in the study and their structures**

| **S.No** | **Compound Name** | **Structure of the compound** |
| --- | --- | --- |
| 1. | Chlorogenic Acid | 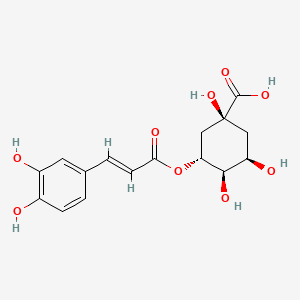 |
| 2. | Ellagic Acid | 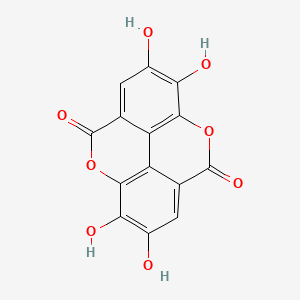 |
| 3. | Curcumin | 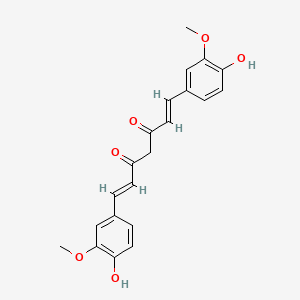 |
| 4. | Ferulic acid | 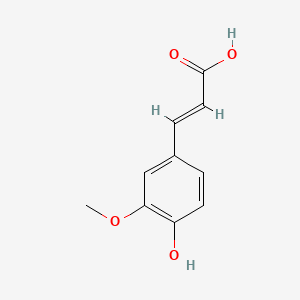 |
| 5. | Kaempferol | 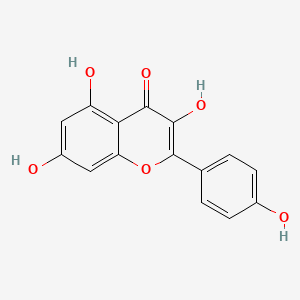 |
| 6. | Genistein | 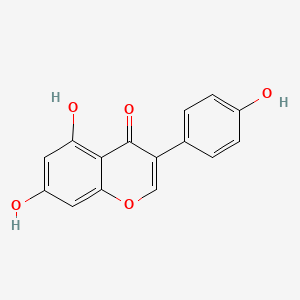 |
| 7. | Lignan | 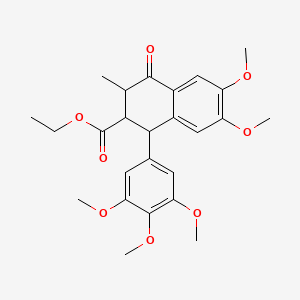 |
| 8. | Luteolin | 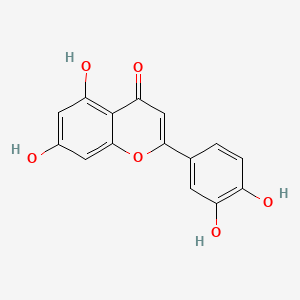 |
| 9. | Naringenin | 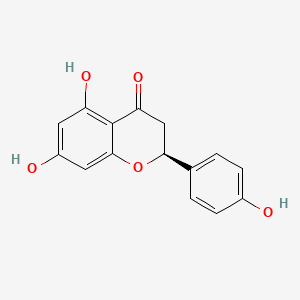 |
| 10. | Quercetin | 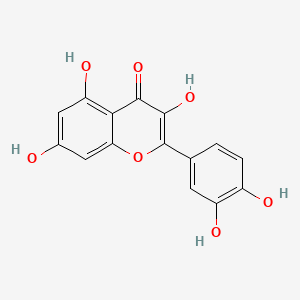 |
| 11. | Resveratrol | 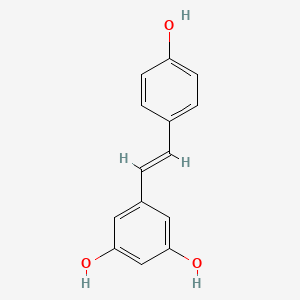 |
| 12. | Rottlerin | 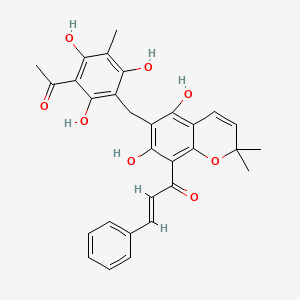 |
| 13. | Rutin | 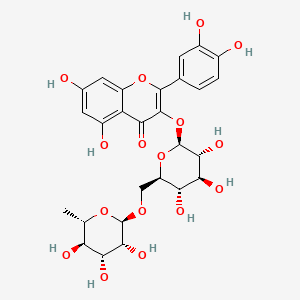 |
| 14. | Silymarin | 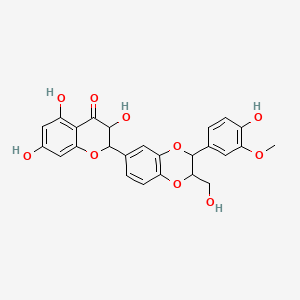 |
| 15. | Apigenin | 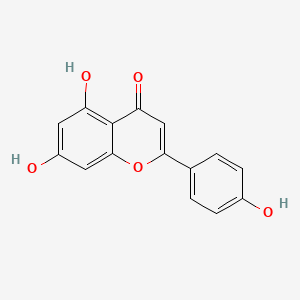 |
| 16. | Berberine | 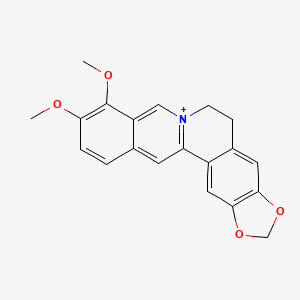 |
| 17. | Pterostilbene | 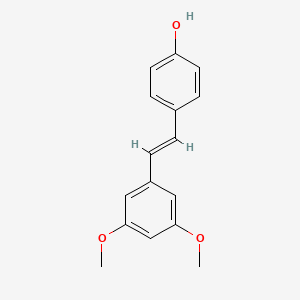 |
